# Sudden cardiac death in the Kazakh and Han peoples of Xinjiang, China: A comparative cross-sectional study

**DOI:** 10.1097/MD.0000000000018126

**Published:** 2019-12-16

**Authors:** Jianghua Zhang, Xianhui Zhou, Qiang Xing, Yaodong Li, Ling Zhang, Qina Zhou, Yanmei Lu, Meiling Zhai, Jianfu Bao, Baopeng Tang

**Affiliations:** a First Affiliated Hospital of Xinjiang Medical University, Urumqi; b Xinyuan People's Hospital, Xinyuan; c Barkol People's Hospital, Barkol, Xinjiang Uygur Autonomous Region, China.

**Keywords:** epidemiological survey, Han, Kazakh, sudden cardiac death, Xinjiang

## Abstract

Sudden cardiac death (SCD) is a major cause of mortality in China. This study collected reference data for future programs of prevention of SCD among the ethnic Kazakh and Han populations in Xinjiang, China.

From January 1, 2015 to December 31, 2015, 2 monitoring locations in northern Xinjiang China were utilized. These locations were selected based on the geographic, economic, and administrative structures of the ethnic Kazakh settlements in Xinjiang. Investigators were trained to investigate SCDs in Kazakh and Han people, a study population totaling more than 400,000. The populations were compared for SCD incidence.

The average age of the Han population was significantly higher than that of the Kazakh. During the year 2015, there were 135 SCDs, specifically 67 and 68 in the Han and Kazakh populations, respectively, incidences of 37.94 and 36.2 per 100,000. After standardizing for age, the incidence in these populations was 29.36 and 51.85 per 100,000. Among those who experienced SCD, the prevalence of hypertension was higher in the Kazakh group than in the Han. The multivariate analysis of populations with SCD showed that, among the patients with coronary heart disease, the Kazakh were more likely to have SCD than the Han (odds ratio: 3.58, confidence interval: 1.18–10.95).

Among the elderly, the incidence of SCD was much higher in the Kazakh population than in the Han population. Basic medical services and health education should be strengthened in the Kazakh pastoral areas.

## 1 Introduction

Sudden cardiac death (SCD) is unexpected death with a cardiac cause, occurring within 1 hour after the onset of acute symptoms. SCD is characterized by sudden onset, and sometimes without any clear prodromal symptoms.^[1,2]^ In many cases, SCD is the first clinical symptom and usually occurs outside the hospital, and in most cases, an equipped and timely ambulance is not available.

Almost 50% of all cardiac deaths are sudden.^[3]^ In the United States, 300,000 to 350,000 people suffer SCD annually.^[4]^ China conducted a nationwide survey on SCD in 2005, and found 41.8 SCDs per 100,000 people for the year. In China, it is estimated that there are more than 500,000 SCDs annually.^[5]^

The Xinjiang Uygur Autonomous Region is located at the northwestern border of China. It is China's largest geographic province, with 1.66 million square kilometers and a population of more than 23 million. The region is economically underdeveloped and multiethnic, with Uygur, Han, Kazakh, Hui, Mongolian, and 47 other ethnic groups.^[6]^ The third-largest ethnic group in Xinjiang is the Kazakh. Approximately 1.6 million Kazakhs live in Xinjiang, primarily in the northern region (ie, north of the Tian Shan mountain range). Most Kazakhs are engaged in animal husbandry.

The prevalence of cardiovascular disease in the Kazakh differs from that of other ethnic groups in Xinjiang, particularly with respect to the prevalence of hypertension, which is significantly higher compared with other ethnic groups.^[7,8]^ In our previous epidemiological survey, Kazakhs were found to have a higher risk of SCD compared with the Han.^[9]^ In the present study, we utilized epidemiological data collected during the year 2015 to investigate the characteristic features of SCDs in the Kazakh population, and compared these with the Han population of the same region. Our data and analysis should serve as references for implementing policies to prevent SCDs among different ethnic groups.

## 2 Methods

### 2.1 Site selection for epidemiological survey

In China, members of the Kazakh ethnic group live predominantly in the northern mountains, while the urban population is relatively low. Most Kazakh people are engaged in animal husbandry. Some Kazakh herdsmen are nomadic. Our sampling area took into consideration a range of factors, including the geography, economy, population, and ethnicity of the target study group.

Xinyuan County and Barkhun County, which are located to the west and east of northern Xinjiang, respectively, were selected as the monitoring sites for the present study. These 2 counties have the highest concentration of Kazakh people, accounting for more than half of the total population. Here, they are mainly engaged in animal husbandry. The overall level of education of the Kazakhs is low. In particular, older people have higher illiteracy rates and are less proficient in Chinese and Chinese characters. The per capita gross domestic product of the 2 counties is approximately 5000 to 10,000 renminbi (RMB).

### 2.2 Data source

China has a household registration (Hukou) system. The household registration authority establishes every citizen's account at birth, manages the account, and the account is canceled at death. Investigators from the household registration authority visit the police stations of each street or village in their area every quarter, to obtain the death certificate of those who died, and fill in the death questionnaire. If the patient died in the hospital, or when medical staff is involved at the time of death, investigators access the relevant information from the hospital, to fill in the death questionnaire.

If the patient died outside the hospital and no medical staff was present, no relevant medical information may be available. In these cases, investigators contact witnesses of the death or relatives of the deceased patient to obtain relevant information and to fill in the death questionnaire. The death questionnaire is then reviewed by the person in charge of each monitoring site to determine if the death was SCD.

### 2.3 Training of investigators and quality control

The project team established a cooperative agreement with the county hospitals of the 2 monitoring locations before the survey began. At each monitoring location, a physician with the title Deputy Director of Cardiology was designated the head of that location. A physician with a grade of residency higher than 1 was assigned as the investigator, and this person received project and epidemiological survey training at our hospital.

To ensure the comprehensiveness of the epidemiological survey, the investigation of deaths was not limited to the household registration system. Surveyors collected data regarding deaths from the household registration authorities every quarter, but also investigated possible omissions or delayed death reports at the community and township level.

The diagnostic criteria for SCD are a heart-related death with a sudden loss of consciousness that occurs within 1 hour after the onset of acute symptoms. In addition, deaths of people with no clear history of heart disease, no symptoms before bedtime, and who died during sleep were also classified as SCD. The person in charge at the monitoring location determined whether the case was an SCD based on the available information, and sent the data to our center every quarter.

The project team consisted of 4 chief cardiovascular physicians, who were responsible for the final review of a diagnosis of SCD. If the project team was in doubt about a particular case, the questionnaire was returned to the investigator at the monitoring site and the investigator was asked to supplement key data points. Each quarter, investigators from our center compared the data pertaining to SCD cases with that of the Centers for Disease Control to determine if any cases were missed. If a case of suspected SCD was missed, the person's information was sent to the regional monitoring location, and the investigators were asked to review and clarify the cause of death.

### 2.4 Diagnosis of heart disease

The diagnosis of basal heart disease in patients with SCD is based on information from the patient's previous medical records. In addition, information on past medical history obtained from the family of the deceased is used as a reference. The main diagnostic criteria for heart disease are as follows.

For a diagnosis of coronary heart disease, the patient's medical records clearly indicated coronary heart disease, and the patient was taking drugs to treat coronary heart disease. A previous coronary angiography or coronary computer tomography could also clearly indicate coronary heart disease. In addition, the patient may have had a clear history of myocardial infarction but no symptoms of heart failure, and was taking medication for coronary heart disease.

Hypertension was considered when a patient's medical record clearly stated a diagnosis of hypertension, or contained records of blood pressure values consistent with hypertension, or the patient was prescribed medication to treat hypertension. Cardiac valve disease was defined as a medical history clearly indicating cardiac valve disease; the physical examination conformed to the characteristics of cardiac valve disease, and cardiac ultrasound confirmed cardiac valve abnormality.

Ischemic cardiomyopathy was diagnosed when the patient's medical history clearly indicated ischemic cardiomyopathy, or a history of myocardial infarction, symptoms of cardiac insufficiency, echocardiography findings that indicated ischemic cardiomyopathy with decreased ejection fraction, and the patient had been taking anti-heart failure drugs. A diagnosis of dilated cardiomyopathy depended upon medical history data that clearly indicated dilated cardiomyopathy, symptoms of cardiac insufficiency, echocardiography findings showing dilated cardiomyopathy with decreased ejection fraction, and the patient had been taking anti-heart failure drugs.

Patients with congenital heart disease had medical history data that clearly indicated congenital heart disease, and echocardiography findings were in accordance with congenital heart disease. Pulmonary heart disease was recorded when the patient's medical history clearly indicated pulmonary heart disease, a long history of chronic obstructive pulmonary disease, and symptoms of right ventricular dysfunction such as lower extremity edema. In addition, cardiac ultrasound suggested an increase in right ventricle and pulmonary hypertension.

Finally, the death was recorded as due to unknown causes when the deceased had no clear history of heart disease and lacked medical records that could be effectively used to determine a clear cause of death.

### 2.5 Statistical methods

Statistical analyses were performed using SPSS 22.0 software. Quantitative variables are described as mean ± standard deviation, and the qualitative variables as frequency. Sampling rates were compared using the Chi-squared test and the Fisher exact test. The *Z*-test was used to compare crude SCD morbidity. Age-adjusted rates were computed by the direct method with the local populations sorted by the following age groups (as of 2014): <18, 18–35, 36–65, and >65 years. A logistic regression model was used for multivariate analysis of the data. A *P* value < .05 was considered statistically significant.

### 2.6 Patient and public involvement statement

The patients and the public were not involved in our study.

### 2.7 Ethics approval

The study protocol was approved by the Ethics Committee of the First Affiliated Hospital of Xinjiang Medical University, China (20150130-01). This study was performed in accordance with the standards of the Ethics Committee and with the 1964 Helsinki declaration and its later amendments or comparable ethical standards.

## 3 Results

### 3.1 Sampling of regional and demographic characteristics

The Xinyuan and Barkol counties are located in the eastern and western regions of northern Xinjiang, respectively. The populations of both counties are dominated by the Han and Kazak ethnic groups. The Han population is principally engaged in agricultural production, while the livelihood of the Kazakh people mainly relies on livestock husbandry.

In this prospective study, from January 1, 2015 to December 31, 2015, we monitored 431,130 people (Table 1). Among them, there were 176,616 Han and 187,867 Kazakh. Overall, there were 1998 deaths. At each sampling location, the gender ratio of the Han and Kazakh was similar. However, the average age of the Han population was higher than the average age of the Kazakh, and there were twice as many Han people older than 60 years as there were in the Kazakh population.

**Table 1:**
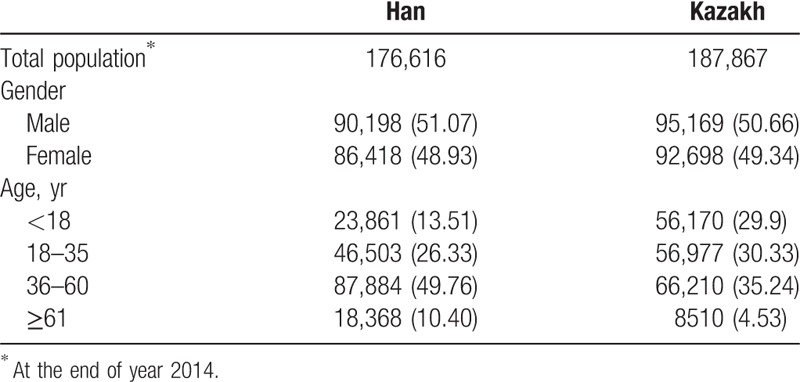
Demographics of the Han and Kazakh ethnic populations, n (%).

### 3.2 SCD

During the 1-year monitoring period, there were 135 SCDs in the Han and Kazakh populations, specifically 67 (49.63%) and 68 (68.3%), respectively (Table 2). The SCDs of the Han and Kazakh populations were statistically comparable with regard to gender ratio and average age at time of death. Specifically, among the Han, there were 43 (64.2%) men and 24 (35.8%) women who suffered SCD. Among the Kazakh, there were 40 (58.8%) men and 28 (41.2%) women with SCD. The average age at SCD in the Han population was 61.73 ± 13.06 years, while that of the Kazakh population was 62.26 ± 15.98 years. The 2 populations were also similar with regard to the number of SCDs per 100,000 people: 37.94 in the Han, and 36.20 in the Kazakh. However, after standardization for age, the incidence of SCD was significantly higher in Kazakhs than in Hans, with 51.85 deaths compared with 29.36 deaths per 100,000 (Table 2).

**Table 2:**
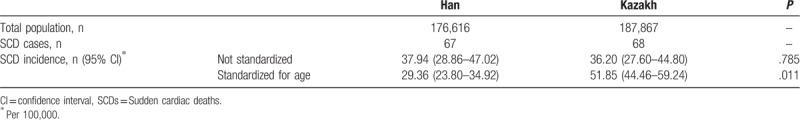
SCDs in the Han and Kazakh populations, n.

In the Han population, the incidences of SCDs per 100,000 in men and women were 47.67 and 27.77, respectively, while in the Kazakh these rates were 42.03 and 30.21 per 100,000. Thus, in both ethnic groups the incidence of SCD was higher in men than in women.

In people younger than 60 years, the rates of incidence of SCDs of the Han and Kazakh were statistically similar. However, among individuals older than 60 years, the incidence rate of SCD was significantly lower in the Han population than in the Kazakh (Table 3).

**Table 3:**
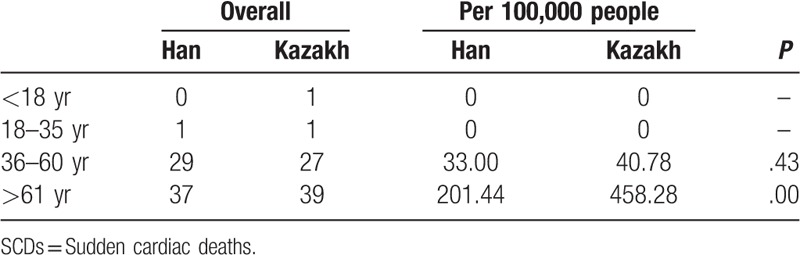
SCDs in the Han and Kazakh ethnic groups by age group, n.

### 3.3 Heart diseases in people with SCD

Coronary heart disease was the most common underlying cardiovascular disease in both ethnic groups (Table 4). Among the people who experienced SCD, in the Kazakh population the prevalence of coronary heart disease and hypertension was lower and higher, respectively, compared with the Han.

**Table 4:**
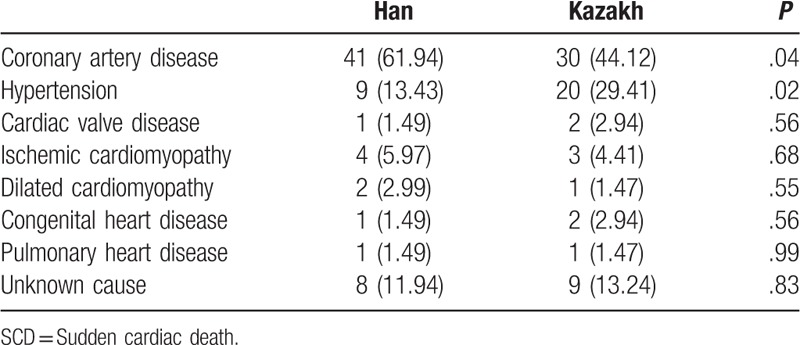
Underlying cardiovascular diseases in the 2 ethnic groups with SCD, n (%).

### 3.4 Risk factor

The age, gender, and existence of underlying diseases in the 2 SCD groups were used in the multivariate unconditional logistic regression model and analyzed by stepwise regression. Kazakh patients with coronary heart disease were found more likely to die of SCD than Han patients with coronary heart disease (odds ratio: 3.59, confidence interval: 1.18–10.95; Table 5).

**Table 5:**
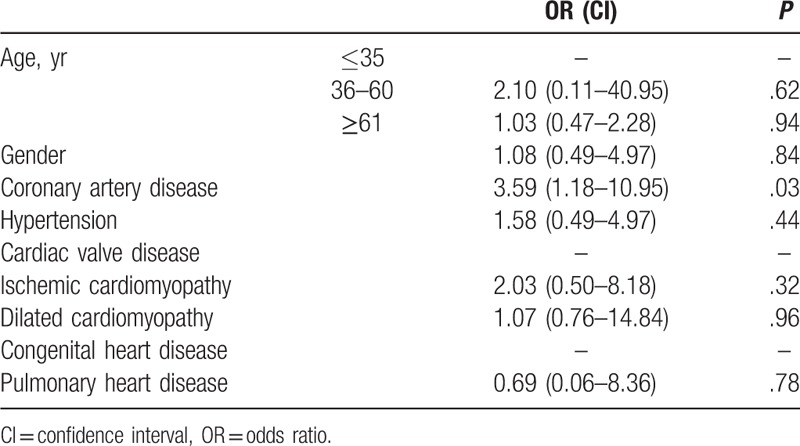
Multi-factor analysis.

## 4 Discussion

Xinjiang has a vast territory and a complex ethnic composition. There are differences in lifestyles and religious beliefs among the different ethnic groups. Economic development is also not homogenous in Xinjiang. The available data suggest that there are differences in the incidences of cardiovascular diseases between the ethnic Han majority and ethnic minorities, and amongst the ethnic minorities. For example, the incidence of peripheral arterial disease is lower among the Kazakh population than the Uyghur,^[10]^ while heart failure is less prevalent among the Han population than the Kazakh.^[7,11]^ Our present epidemiological investigation of SCD in the Han and Kazakh peoples is the first to consider the geographic and ethnic factors of the Xinjiang region.

This study found that the rate of SCD in the Han and Kazakh populations in the year 2015 was similar, but after standardization for age, the incidence was significantly higher in the Kazakh than in the Han. An important reason for this is that the percentage of elderly in the Han is significantly higher than in the Kazakh. In Xinjiang, there are differences in how the family planning policy is implemented between the Han and ethnic minorities, which are allowed 1 and 2 children, respectively. The family planning policy is also more lenient among the ethnic minorities. In rural areas, almost every family of an ethnic minority has more than 3 children. A direct result of these differences, imposed more than 30 years ago, is that the aging population is higher in the Han than in other ethnic groups in Xinjiang.

In the sixth census in 2010, it was found that in Xinjiang, the elderly population was

12.32% Han and 6.06% Kazakh.^[12]^ In our sample area, of those who were older than 60 years, the number of Han was more than twice the number of Kazakh. After stratifying each population by age group, it was also found that the rate of SCD in the Kazakh group was more than twice that of the Han group. This suggests that the prevention and treatment of underlying diseases that lead to SCD in the elderly Kazakh population requires more attention.

Economic factors strongly influence the rate of SCD in populations.^[5]^ There are differences in the economic development of the Han and Kazakh ethnic groups. The economic structure of the Kazakh is very strong and mainly conforms to the traditional mode of production. Many Kazakh herdsmen continue to live near bodies of water and rely on the ancient nomadic practices of animal husbandry. There is no precise data regarding the per capita income of the Kazakh population at present, but according to a 2012 survey of a Kazakh village, the average annual income of the local Kazakh people was 2608 RMD.^[13]^ According to data from the Autonomous Region Statistics Bureau, the per capita annual income of the rural population in Xinjiang was 6394 RMB.

The most effective way to prevent SCD is to implant an implantable auto-defibrillator, each of which costs almost 100,000 RMB. With Medicare reimbursement, citizens pay 30,000 to 40,000 RMB. Therefore, the relatively backward economic development of the Kazak population has restricted the prevention and treatment of SCD in the Kazakh population to a certain extent.

Many of the Kazakh people live in pastoral areas. Due to limited medical resources and lack of basic medical care, many diseases cannot be effectively treated. This is also an important reason for the higher overall death rate among the Kazakh people in Xinjiang, compared with other ethnic groups. Furthermore, due to the low education level of elderly Kazakhs living in pastoral areas, disease awareness is low and medical compliance is poor. Previous studies have reported that hypertension develops earlier in the Kazakh people compared with the Han, but rates of awareness and treatment are significantly lower.^[14]^

Coronary heart disease accounts for approximately half of all underlying cardiac diseases. In counties with Han and Kazak populations, coronary heart disease is the major underlying cardiac disease leading to SCD. Previous studies conducted in China and elsewhere have been consistent in these findings.^[5,15]^ However, there are differences between the Han and Kazak ethnic groups. Among those who suffer SCD, the rate of coronary heart disease is lower among the Kazakh, while the prevalence of hypertension is higher, compared with the Han population. However, Kazakh patients with coronary heart disease were found to be more likely to die of SCD than Han Chinese patients with coronary heart disease. These disease rates in SCD correlate with the rates in the general Kazakh population. For example, the percentage of Kazakh with hypertension and heart failure is higher than that of other ethnic groups.^[8,16,17]^ Notably, the rate of type 2 diabetes in the Kazakh is lower than that of the Uyghurs.

Compared with developed regions of China,^[18]^ there is still a relatively high incidence of SCD. In addition, pastoral lifestyles are also a possible cause of high rates of hypertension. Previous studies on cardiovascular events in Xinjiang have found that the prevalence of hypertension in pastoral populations is significantly higher than in urban populations.^[19]^ Therefore, the prevention and treatment of Kazakh cardiovascular disease can have a lower incidence of Kazakh SCD.

In our study, SCD was defined according to the World Health Organization as unexpected death with a cardiac cause, occurring within 1 hour after the onset of acute symptoms. However, according to an autopsy study,^[20]^ this definition may result in a false positive misdiagnosis, and; therefore, the actual rate of SCD in the present study may be lower than what we determined. Unfortunately, few families of the deceased would agree to an autopsy, as autopsies are not performed unless there is a legal mandate. Therefore, no autopsies were performed in this study. In addition, due to poor medical conditions in remote areas and that most of the sudden deaths occurred outside the hospital, the detailed clinical data and the patient's electrocardiographic performance during sudden death were lacking, which may present some defects in the diagnosis of SCD.

The high incidence of SCD in the Kazak is the result of a combination of factors, including economic, demographic, lifestyle, and cost of health insurance. More measures should be taken to improve the healthcare system in pastoral regions and health education for the Kazakh population, to increase their awareness of cardiovascular disease and subsequently improve treatment compliance. Only by comprehensively improving the prevention and treatment of basic cardiovascular diseases can we effectively reduce the incidence of SCD in the Kazakh population. In formulating preventive strategies, clinicians should pay more attention to prevention and control of hypertension in this population.

## Author contributions

**Data curation:** Qiang Xing, Yaodong Li, Ling Zhang, Qina Zhou, Yanmei Lu, Meiling Zhai, Jianfu Bao.

**Formal analysis:** Qiang Xing, Yaodong Li, Ling Zhang, Qina Zhou, Yanmei Lu, Meiling Zhai, Jianfu Bao.

**Project administration:** Jianghua Zhang, Baopeng Tang.

**Writing – original draft:** Jianghua Zhang.

**Writing – review and editing:** Xianhui Zhou, Baopeng Tang.
